# A Two-Stage Enzymolysis Method and Its Application in Exerting Antioxidant Activity of Walnut Protein

**DOI:** 10.3389/fnut.2022.889434

**Published:** 2022-04-14

**Authors:** Dandan Liu, Min Chen, Junsong Zhu, Weijie Tian, Yiting Guo, Haile Ma

**Affiliations:** ^1^School of Food and Biological Engineering, Jiangsu University, Zhenjiang, China; ^2^Institute of Food Physical Processing, Jiangsu University, Zhenjiang, China; ^3^Laboratory Animal Research Center, Jiangsu University, Zhenjiang, China

**Keywords:** walnut protein, antioxidant activity, *in vitro* limited enzymolysis, *in vitro* simulated gastrointestinal digestion, *in vivo* verification

## Abstract

Traditional enzymolysis method for producing bioactive peptides does not consider the utilization of digestive enzymes in the human gastrointestinal tract, leading to the possibility of excessive hydrolysis and higher production cost. Therefore, a two-stage enzymolysis method was established in this study based on *in vitro* limited enzymolysis and gastrointestinal digestion, and applied it to the research of walnut protein (WP) in exerting antioxidant activity. Results showed that WP could be well-digested by pepsin and pancreatin. WP with limited enzymolysis degree of 0% could achieve high antioxidant activity after the simulated gastrointestinal digestion, and the 2,2-Diphenyl-1-picrylhydrazyl (DPPH) scavenging activity and reducing power were 66.53% and 8.55 μmoL TE/mL, respectively. *In vivo* experimental results also exhibited that both WP and WP hydrolysate (WPH) could alleviate the oxidative damage induced by D-galactose in SD rats to some extent. Considering the digestive function of human body, *in vitro* limited enzymolysis, *in vitro* simulated gastrointestinal digestion and *in vivo* validation are necessary processes for the production of bioactive peptides.

## Introduction

Bioactive peptides are functional compounds composed of 2–15 amino acid residues, which are mainly released from the inactive form of the precursor protein by enzymatic hydrolysis, microbial fermentation, chemical extraction, and *in vitro* simulated gastrointestinal digestion or *in vivo* digestion (1, 2). Enzymatic hydrolysis is the most widely used method in the production of bioactive peptides to improve the nutritional value of proteins (3). However, traditional preparation technology of bioactive peptide was mainly based on the theory that the small molecular peptides can be directly absorbed after entering the gastrointestinal tract. The peptides with high bioactivity *in vitro* were screened directly without considering the function of digestive enzymes in the gastrointestinal tract after intaking the peptides. Literature has shown that the gastrointestinal tract is a major barrier for the oral administration of bioactive peptides due to the impact of the digestive enzymes and extreme pH conditions to the structure and bioactivity of peptides (4). The formed bioactive peptides may be excessively hydrolyzed by the digestive enzymes in the gastrointestinal tract, leading to the destruction of the original structure and reducing its bioactivity. In order to maintain the bioactivity of the antioxidant hydrolysates and peptides after gastrointestinal digestion, the additional and laborious operation such as screening bioactive peptides with digestive stability (5) and the encapsulation technology (6) were used in some studies to prevent the excessive digestion of the peptide during the gastrointestinal digestion.

**Figure 1:**
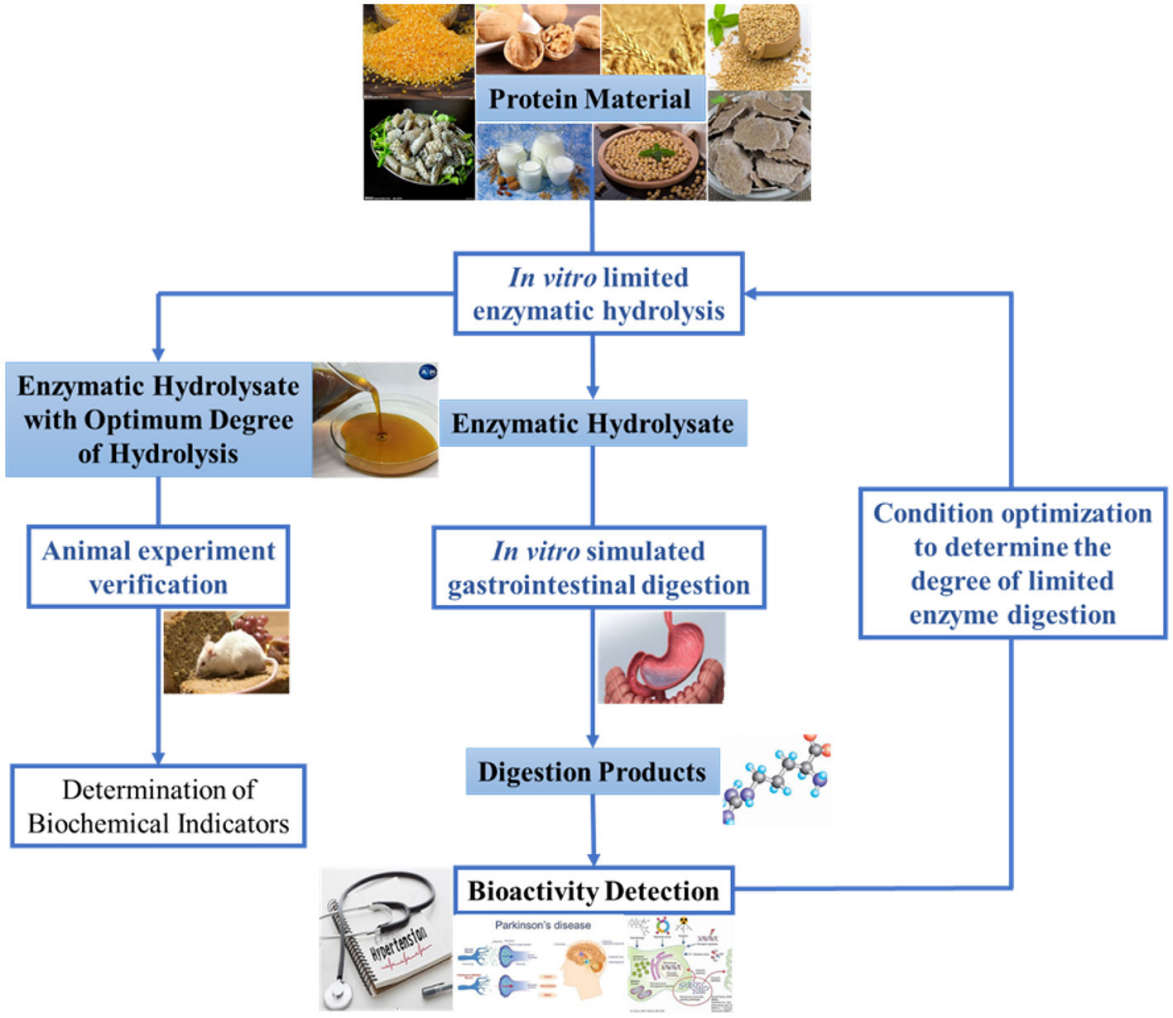
Schematic representation of two-stages enzymatic hydrolysis method based on *in vitro* limited enzymatic hydrolysis and *in vivo* digestion.

A proper method of enzymatic hydrolysis for preparing bioactive peptides ought to include two stages: *in vitro* limited enzymatic hydrolysis and *in vivo* gastrointestinal digestion. *In vitro* limited enzymatic hydrolysis means that the protein material is partially enzymolyzed into macromolecular peptides by commercial proteases *in vitro*. After the macromolecular peptides were intaked by the human body, the digestive enzymes in the human gastrointestinal tract are utilized for further enzymolysis, and finally small molecular peptides with certain bioactivity are obtained in the gastrointestinal tract. Due to the cumbersome and costly digestion process *in vivo*, this study first adopted simulated gastrointestinal digestion combined with *in vitro* limited enzymatic hydrolysis to determine the optimal degree of hydrolysis (DH) *in vitro*, and then carried out *in vivo* verification. Compared with the traditional method, the two-stage enzymatic hydrolysis method is characterized by moving the activity detection node rearward to after gastrointestinal digestion process. This method has the following advantages: (i) make good use of the digestive enzymes and enzymolysis environment of the human gastrointestinal tract; (ii) prevent excessive enzymatic hydrolysis of gastrointestinal digestive enzymes from destroying the structure and function of bioactive peptides; (iii) reduce the amount of protease for *in vitro* enzymatic hydrolysis, shorten the time and reduce the cost of *in vitro* enzymatic hydrolysis. The degree of *in vitro* limited enzymolysis is depended by the structure, amino acid composition and digestibility of the protein. Some proteins may require thorough *in vitro* enzymolysis, some proteins may require partial *in vitro* and partial *in vivo* enzymolysis, while some may require only *in vivo* digestion to exert bioactivity. According to this principle, our research group proposed that the production of bioactive peptides by enzymatic hydrolysis should be carried out in the following stages: (i) *in vitro* enzymatic hydrolysis; (ii) *in vitro* simulated gastrointestinal digestion; (iii) bioactivity detection; (iv) animal experiment verification (as described in Figure 1).

Walnut protein (WP) is an excellent plant protein resource with good balance of essential amino acids (7). As a kind of nut protein, WP shows a high digestibility. Zhang et al. (8) reported that the digestibility of WP could reach 74–90% *in vitro* and 87.02% *in vivo*. In another study, Szemilao and Sathe (9) showed that walnut glutelins was highly digestible proteins *in vitro*. The WP hydrolysates and its isolated peptides have been proven to have excellent antioxidant activities and health benefits. Feng et al. (1) reported that the antioxidant peptides Thr-Tyr and Ser-Gly-Gly-Tyr isolated from walnut proteolysis showed a strong 2'-azinobis-(3-ethylbenzthiazoline-6-sulphonate) (ABTS) radical scavenging ability and high oxygen radical absorbance capacity (ORAC) value, and Ser-Gly-Gly-Tyr also exhibited a good protective effect on cells against oxidative damage. Ren et al. (7) found that the walnut proteolysate with molecular weight <3 kDa not only had good antioxidant activities, but also attenuated memory impairment in mice. However, these studies on the production of antioxidant peptides derived from WP were mainly based on the traditional theory of direct utilization of small molecule peptides, without considering the actual utilization of antioxidant peptides *in vivo*.

Therefore, this study explored the application of the two-step enzymatic hydrolysis method in exerting antioxidant activity of WP. In detail, five commercial proteases were used for the limited enzymatic hydrolysis of WP to produce WP hydrolysates (WPH) with different DH, and then performed *in vitro* simulated gastrointestinal digestion. The peptide content, 2,2-Diphenyl-1-picrylhydrazyl (DPPH) radical-scavenging activity and reducing power of gastrointestinal digests were used to determine the optimal DH of *in vitro* limited enzymatic hydrolysis. Moreover, the protective effects of WPH with the optimal DH against oxidative stress in the D-galactose-induced rats were evaluated.

## Materials and Methods

### Materials

WP was provided by Changbai Fairy Biotechnology Co., Ltd. (Liaoning, China), which contained 75% protein (dry weight). Alcalase (350,507 U g^−1^), neutrase (230,453 U g^−1^) and pancreatin (109,000 U g^−1^) were obtained from Novozymes Co., Ltd. (Nanjing, China). Pepsin (32,600 U g^−1^), papain (21,413 U g^−1^), flavourzyme (64,144 U g^−1^), protamex (226,186 U g^−1^), DPPH and D-galactose were purchased from Sigma-Aldrich (St. Louis, MO, USA).

### Preparation of WPH Based on *in vitro* Limited Enzymatic Hydrolysis

The WPH was prepared by different proteases according to our previous study (10). Briefly, WP was mixed with distilled water in a ratio of 1:20 (w/v), and then the mixture was stirred and adjusted to the optimum temperature and pH of proteases. The WP was hydrolyzed under the catalysis of different protease (5,000 U/g, of protein), and the pH of the reaction system was maintained constant with 0.5 M NaOH. Enzyme was inactivated by keeping the reaction system in boiling water for 10 min after hydrolysis. The supernatant of the hydrolysate was collected after centrifuged (TGL18M, Kate Laboratory Instrument Co., Ltd, Yancheng, China) at 11,000 rpm for 10 min.

### Determination of the DH

The DH was measured according to the pH-stat method (11). The DH was calculated following the equations below:


(2.1) $$\begin{array}{r} DH\left(\%\right)=\frac{h}{h_{tot}}*100=\frac{B*N}{\alpha *mh_{tot}}*100 \end{array}$$

(2.2) $$\begin{array}{r} \alpha =\frac{10^{pH-pK}}{1+10^{pH-pK}} \end{array}$$

where *B* is the consumed volume of 0.5 M NaOH (mL); *N* is the concentration of NaOH (M); α is the average degree of dissociation of the α-NH_2_ groups; *m* is the mass of hydrolyzed protein (g); and *h*_*tot*_ is the total number of peptide bonds per gram of protein, which is 8.0 mmol g^−1^ for WP (12).

### Simulated *in vitro* Gastrointestinal Digestion

Simulated *in vitro* gastrointestinal digestion was conducted as described in our previous work (13). Briefly, the samples were mixed with pepsin (2%, w/w of protein) and incubated under the gastric condition (37°C, pH 1.5) for 2 h. Subsequently, after the simulated gastric digestion, pancreatin was added (2%, w/w of protein) to the mixture to continue the simulated intestinal digestion under the intestinal condition (37°C, pH 7.5) for 4 h. Finally, the supernatants of the WP and WPH digests were collected after the reaction system was inactivated.

### Determination of Peptide Content

Peptide contents were determined by the Folin phenol method as described by Yang et al. (11).

### *In vitro* Antioxidant Activity Assay

#### DPPH Radical-Scavenging Activity

As depicted in our previous study (10), a proper amounts of 0.2 mM DPPH ethanol solution were mixed with and sample in equal volumes, and then kept in the dark at 37°C for 30 min. The absorbance was determined by the spectrophotometer (Unic 7200, Unocal Corporation, Shanghai, China) at 517 nm. The control was set up using equal volume ethanol instead of the sample. The DPPH scavenging activity was calculated as follows:


(2.3) $$\begin{array}{r} DPPH\text{ radical scavenging activity }\left(\%\right)=\frac{A_{0}-A_{1}}{A_{0}}*100 \end{array}$$

where *A*_0_ and *A*_1_ are the absorbance of the control and the sample, respectively.

#### Reducing Power

The reducing power was measured according to the method described by Zheng et al. (14). Two milliliters of sample were mixed with 2.0 mL of 0.2 M sodium phosphate buffer (pH 6.6) and 2.0 mL potassium ferricyanide (1%, w/v). After incubating the mixture at 50°C for 20 min, 1.0 mL trichloroacetic acid (10%, v/v) was added and then centrifuged at 3,000 rpm for 10 min. Two milliliters of the supernatant were collected and mixed with 2.0 mL of distilled water and 0.4 mL of 0.1% (w/v) FeCl_3_, and let stand at room temperature for 10 min. The absorbance at 700 nm was measured by a spectrophotometer. The Trolox standards with different concentration were used to plot the standard curve and the reducing power was express as μmol TE (Trolox equivalent) /mL of antioxidant.

### *In vivo* Antioxidant Experiment

#### Animal Experiment Design

Forty healthy 6-week-old sprague dawley (SD) rats (male; 200 ± 20 g body weight, BW) were provided by the Laboratory Animal Research Center of Jiangsu University (License number: SCXK (Jiangsu) 2018-0012, certificate number: 201932819). SD rats were raised in a standard sterile barrier system at 20–26°C and 40–70% humidity, and daily light-dark cycle of 12 h throughout the experimental period. All experiments were reviewed and approved by the Institutional Animal Care and Use Committee of Jiangsu University (No. 20180053) and performed in accordance with the guidelines of the National Institutes of Health Guide for the Care and Use of Laboratory Animal. After 7 days of acclimatization, the rats were randomly allocated into five groups (*n* = 8), namely Control, D-Gal, WP-I, WP-II, and WPH group with the details of each group showed in Table 1. The SD rats of each group were intraperitoneal injected and oral administered simultaneously once-daily according to the dosages described in Table 1.

**Table 1:** Animal grouping and experimental design.

**Groups**	**Injectable drug**	**Dosages (mg/kg bw)**	**Administerial reagents**	**Dosages (mg/kg bw)**
Control	Saline	0	Sterile water	0
D-gal	D-galactose	200	Sterile water	0
WP-I	D-galactose	200	Walnut protein	400
WP-II	D-galactose	200	Walnut protein	800
WPH	D-galactose	200	Walnut protein hydrolysate	800

*bw, body weight*.

#### Determination of the Growth Rate of BW and Organ Index of SD Rats

The BW of rats was recorded every week during the experiment. The growth rate of BW was calculated as the following equation:


(2.4) $$\begin{array}{r} Weightgrowthrate\;\left(\%\right)={Weightgain/Initialweight}^{*}100\% \end{array}$$

After 6 weeks administration, the SD rats were anesthetized with 1% pentobarbital sodium (3 mL/kg BW). The celiac arterial blood of the rats was collected using the vacuum negative pressure tube, and then stored at room temperature for subsequent use. The heart, liver, spleen, lung and kidney were separated, rinsed and weighed. The organ index was calculated as follows:


(2.5) $$\begin{array}{l} Organ\;index\left(\%\right)=Organ\;weight/Body\;weight*100\% \end{array}$$

#### Histological Assay

The liver tissues of SD rats were fixed in 10% (w/v) formaldehyde, embedded in paraffin and then stained with hematoxylin and eosin (H.E.). The histopathological changes of liver tissues were observed by light microscope (Leica 6500, Germany).

#### Determination of Serum Indexes in Rats

The collected artery blood of SD rats was stored at room temperature for 1 h to coagulate spontaneously, and then the supernatant was obtained by centrifuging (4°C, 3,000 rpm, 15 min). The level of malondialdehyde (MDA), glutathione (GSH), total antioxidant capacity (T-AOC) and superoxide dismutase (SOD) in serum were determined by the microplate spectrophotometer (Tecan Infinite PRO TWIN 200, Tecan, Switzerland) and experimental assay kits (Nanjing Jiancheng Biotechnology Research Institute, Nanjing, China).

### Statistical Analysis

Data were expressed as the means ± standard deviation (SD). The differences were evaluated using the one-way analysis of variance (ANOVA). Statistical analysis was accomplished using SPSS 19.0 (SPSS Inc., USA) software.

## Results and Discussion

### Peptide Content of WP and WPH Before and After *in vitro* Simulated Digestion

**Figure 2:**
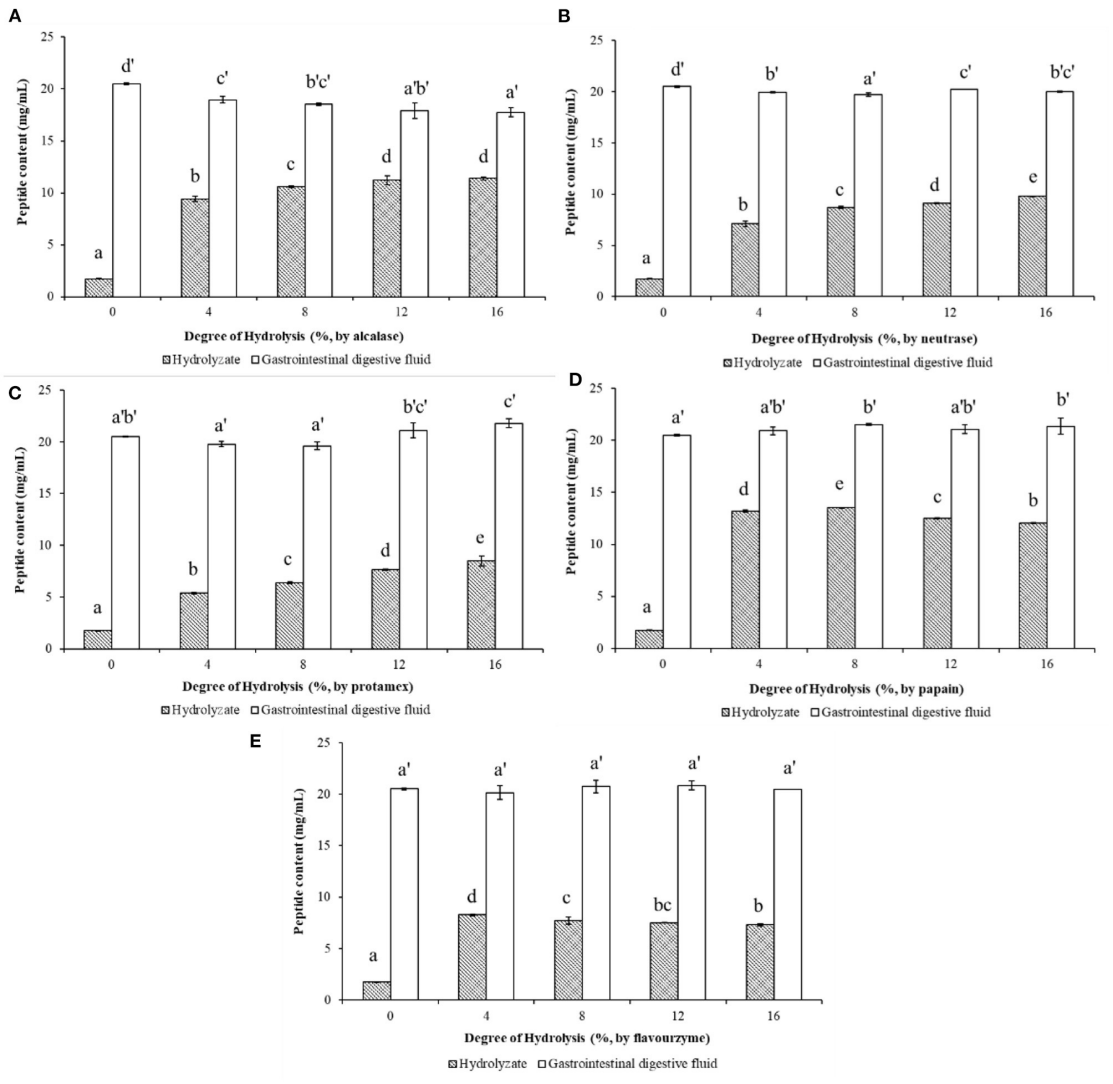
Peptide contents of walnut protein hydrolysates before and after the simulated gastrointestinal digestion *in vitro*. **(A)** Walnut protein hydrolysate produced by alcalase; **(B)** Walnut protein hydrolysate produced by neutrase; **(C)** Walnut protein hydrolysate produced by protamex; **(D)** Walnut protein hydrolysate produced by papain; **(E)** Walnut protein hydrolysate produced by flavourzyme. The results are expressed as mean ± SD (*n* = 3). Means with different superscripts are significantly different (*p* < 0.05).

After limited enzymatic hydrolysis of walnut protein with five different commercial proteases *in vitro*, the peptide content in the hydrolysate increased significantly with the increase of DH (as shown in Figure 2). Among them, the enzymatic hydrolysis efficiency of alcalase (Figure 2A) and papain (Figure 2D) was relatively high, and higher peptide content could be obtained in the hydrolysate under the same DH compared with other proteases. Papain hydrolysate with 16% DH produced the highest content (13.55 mg/mL) of the peptides in the WPH. After the simulated gastrointestinal digestion *in vitro*, the content of peptides in the digests of WP and WPH increased significantly compared to WP and WPH, which reached 20.504 mg/mL in the WP digest and ranged from 17.744 to 21.804 mg/mL in WPH digest. The results illustrate that the peptides in WP can be released by the enzymatic hydrolysis of commercial protease and the digestion of pepsin and pancreatin, and the digestive effect of pepsin and pancreatin on WP was even better than that of commercial protease. This is because hydrophobic amino acids and acidic amino acids are the main components of WP, while chymotrypsin (in pancreatin) decomposes mostly hydrophobic amino acids and pepsin catalyzes primarily the decomposition of acidic amino acids (9). This is also the main reason for the high digestibility of WP. Therefore, the gastrointestinal digestive system should be considered in the preparation and activity evaluation of walnut bioactive peptides.

### Effects of the DH of *in vitro* Limited Enzymatic Hydrolysis on Antioxidant Activity of WP and WPH

**Figure 3:**
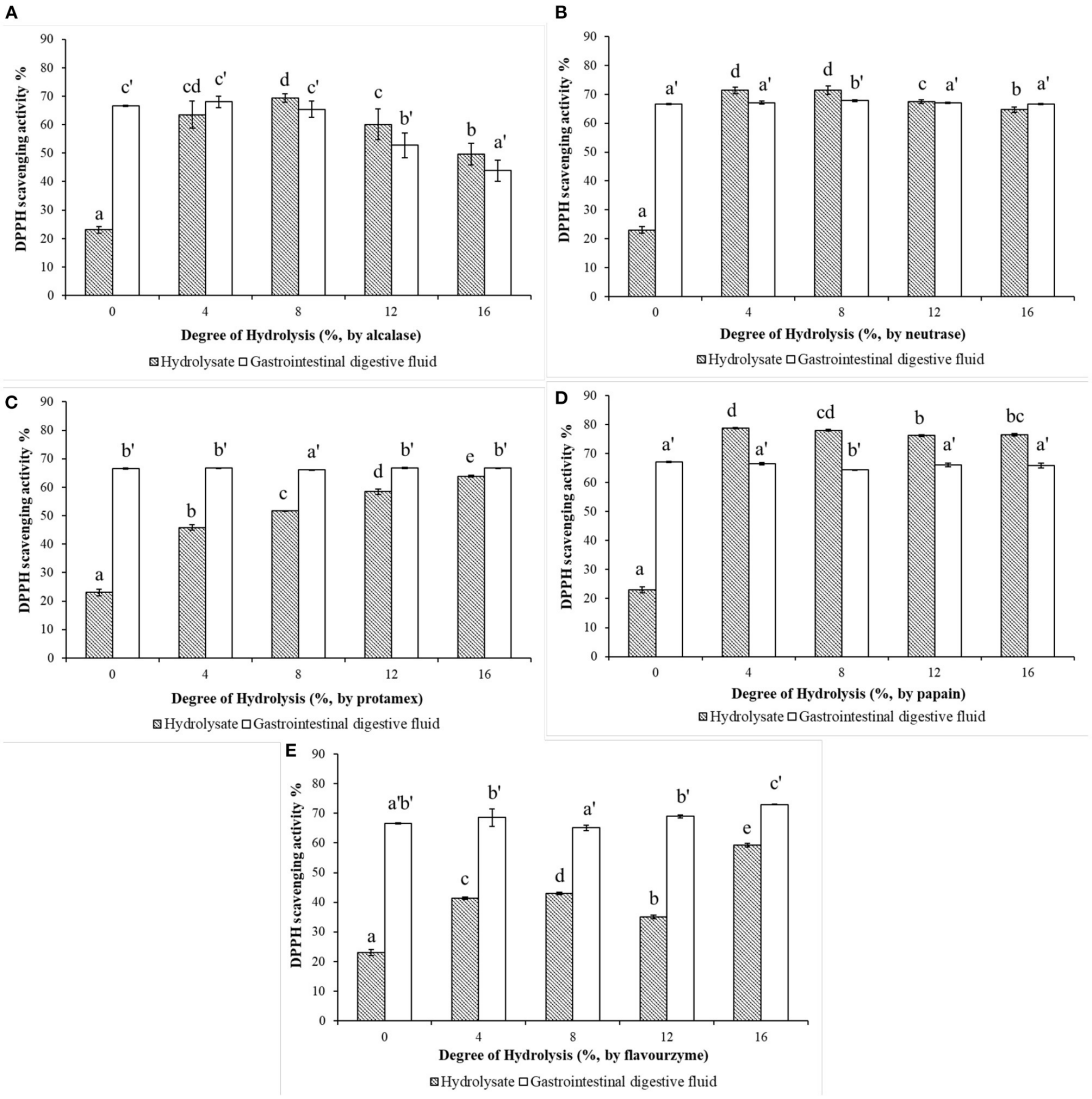
Effects of the degree of hydrolysis (DH) of *in vitro* limited enzymolysis on the DPPH radical-scavenging activity of walnut protein hydrolysates before and after the simulated gastrointestinal digestion *in vitro*. **(A)** Walnut protein hydrolysate produced by alcalase; **(B)** Walnut protein hydrolysate produced by neutrase; **(C)** Walnut protein hydrolysate produced by protamex; **(D)** Walnut protein hydrolysate produced by papain; **(E)** Walnut protein hydrolysate produced by flavourzyme. The results are expressed as mean ± SD (*n* = 3). Means with different superscripts are significantly different (*p* < 0.05).

The DPPH radical-scavenging activities of WP (DH = 0%) and WPH with different DHs after *in vitro* limited enzymatic hydrolysis and their simulated gastrointestinal digests are shown in Figure 3. It can be observed in Figure 3A that, alcalase hydrolysis could enhanced the DPPH radical-scavenging activity of WP. The DPPH scavenging-activity of WPH increased rapidly as the DH changed, reaching the maximum value of 69.39% at DH of 8%, and then decreased. After the simulated digestion *in vitro*, the scavenging-activity of WPH gastrointestinal digestive fluid (WPH-GI) with the DH of 4% reached the highest value of 67.99%, however, which showed no significant difference (*p* > 0.05) when compared to WPH-GI with the DH of 0% (that is WP gastrointestinal digestive fluid, WP-GI). Similar to the results of alcalase hydrolysis, the DPPH scavenging-activity of the neutrase hydrolysate (Figure 3B) increased rapidly as the DH increased, reaching the maximum (71.42%) at the DH of 8%, and then decreased slightly with the continuous increase of DH. When the WPH was digested by pepsin and pancreatin *in vitro*, the DPPH scavenging activity of WPH-GI at DH of 4, 12, and 16% showed no striking difference in comparison with the WP-GI (*p* > 0.05). The enzymatic hydrolysis efficiency of neutrase was high, and it could hydrolyze WP into peptides with high DPPH-scavenging activity under low DH, and the scavenging-activity did not appear to be change significantly with the increase of DH. Meanwhile, the simulated gastrointestinal digestion did not significantly change the DPPH scavenging-activity of WPH, indicating that the peptides with DPPH scavenging activity produced by neutrase hydrolysis had good digestion stability. In the process of protamex hydrolysis (Figure 3C), the DPPH scavenging-activities of WPH showed an upgrade trend as the DH increased (*p* < 0.05). However, the DPPH radical-scavenging activity of protamex hydrolysate was relatively lower than that of alcalase and neutrase hydrolysate at the same DH. After the *in vitro* simulated gastrointestinal digestion, the DPPH radical-scavenging activity of WPH with different DH was improved to varying degrees, and no significant difference (*p* > 0.05) was found between WPH-GI with the DH of 0, 4, 12, and 16%, and slightly higher than WPH-GI with the DH of 8% (*p* < 0.05). Although the DPPH radical-scavenging activity of the protamex hydrolysates was relatively lower than that of other protease hydrolysates under the same DH, both WP and WPH could further improve DPPH radical-scavenging activity to achieve the same effect after the simulated gastrointestinal digestion. Papain had a higher enzymolysis efficiency compared with other proteases used in this work in terms of the DPPH radical-scavenging activity, which was higher than that of other protease hydrolysates under the same DH. The scavenging activity of WPH produced by papain (Figure 3D) reached the maximum value of 78.75% at the DH of 4%, and then decreased slightly as the DH continuously increasing. During the simulated digestion *in vitro*, the peptide bonds and disulphide linkages in WP were broken by pepsin and pancreatin, and resulting a rapid increase in the DPPH scavenging activity. However, the high scavenging activity of papain hydrolysates decreased after *in vitro* simulated gastrointestinal digestion. The highest value of the DPPH scavenging activity (67.11%) was observed in WPH with the DH of 0%, additionally, no significant difference was found between WPH-GI with the DH of 0, 4, 12, and 16% (*p* > 0.05). This is because the WP was enzymatically hydrolyzed into peptides with high DPPH radical-scavenging activity under the catalysis of papain. After the further digestion by pepsin and pancreatin, the original antioxidant characteristic structure of peptides was destroyed and the scavenging activity of DPPH was decreased. The DPPH radical-scavenging activity of WPH was partially improved by flavourzyme hydrolysis (Figure 3E) under the low DH (DH ≤ 12%), and the scavenging activity reached a maximum value of 59.27% when the DH reached 16%. After the *in vitro* simulated gastrointestinal digestion, the DPPH radical-scavenging activity of WPH was further increased (*p* < 0.05), especially the scavenging activity of the WPH-GI with the DH of 16%, which reached 73.01%.

**Figure 4:**
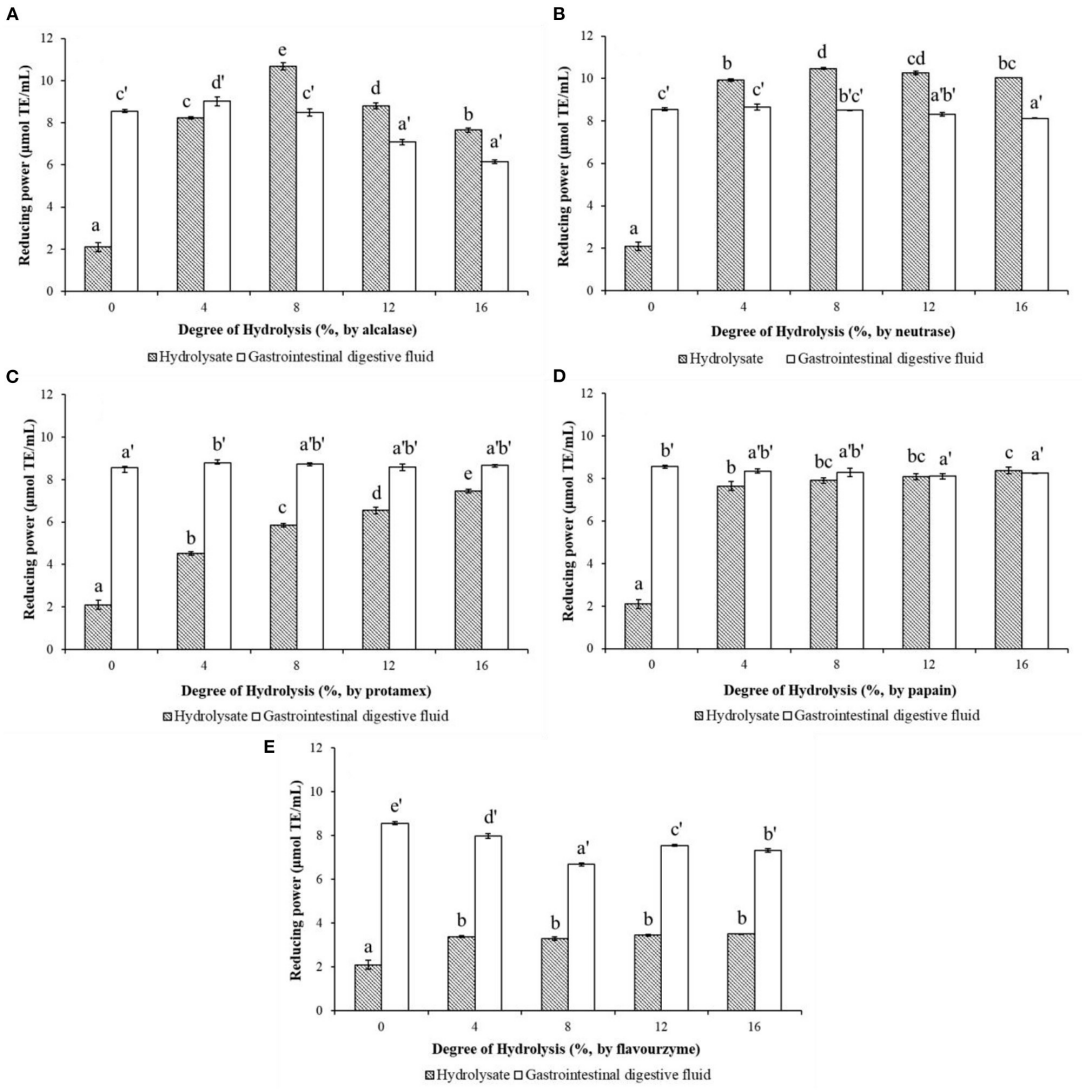
Effects of the degree of hydrolysis (DH) of *in vitro* limited enzymolysis on the reducing power of walnut protein hydrolysates before and after the simulated gastrointestinal digestion *in vitro*. **(A)** Walnut protein hydrolysate produced by alcalase; **(B)** Walnut protein hydrolysate produced by neutrase; **(C)** Walnut protein hydrolysate produced by protamex; **(D)** Walnut protein hydrolysate produced by papain; **(E)** Walnut protein hydrolysate produced by flavourzyme. The results are expressed as mean ± SD (*n* = 3). Means with different superscripts are significantly different (*p* < 0.05).

The reducing power characterizes the ability of a compound to reduce ferric ion (Fe^3+^) to ferrous ions (Fe^2+^) through electrons or hydrogen donation (15). The reducing powers of WPHs produced by different proteases and their *in vitro* simulated gastrointestinal digests are shown in Figure 4. As depicted in Figure 4A, the reducing power of the WPH hydrolyzed with alcalase increased, then dropped a little as DH increase, with the highest value 10.68 μmoL TE/mL at DH of 8%. However, after the *in vitro* simulated gastrointestinal digestion, the reducing power of WPH-GI with the DH of 8% is reduced to 8.48 μmoL TE/mL, which is not significantly different (*p* > 0.05) from that of WP-GI (8.55 μmoL TE/mL). The maximum value (9.02 μmoL TE/mL) of the reducing power occurred in the WPH-GI with the DH of 4%. According to the traditional enzymatic hydrolysis method without considering the function of gastrointestinal digestion, the WPH with the DH of 8% may be selected for further production. However, the WPH with the DH of 4% was the best choice after the evaluation of the two-stages enzymatic hydrolysis method. Similar with the result of the DPPH radical-scavenging activity, the reducing power of neutrase hydrolysate (Figure 4B) increased rapidly with the increase of the DH and reached the highest value of 10.48 μmoL TE/mL at the DH of 8%. After the WPH being digested by pepsin and pancreatin, the reducing power of WPH-GI decreased partly compared with WPH. Finally, the WPH-GI at DH of 4% showed the highest reducing power (8.65 μmoL TE/mL), which had no significant difference (*p* > 0.05) compared with WP-GI (8.55 μmoL TE/mL). The digestion of pepsin and pancreatin could not only hydrolyze WP into WP-GI with high reducing power, but also destroy the active peptides that have been already formed in the WPH, resulting in a decrease in reducing power. When the WP was hydrolyzed by the protamex (Figure 4C), the reducing power of the WPH showed a continuous upward trend as the DH increase, and further increased after the gastrointestinal digestion. Finally, the reducing power of WPH-GI at DH of 4% (8.80 moL TE/mL) was highest, and the reducing power of the WPH-GI with the rest DH showed no significant difference (*p* > 0.05) with that of WP-GI. The reducing power of papain hydrolysates with different DH, and their simulated gastrointestinal digests are depicted in Figure 4D. Due to the high enzymatic hydrolysis efficiency, the hydrolysis of papain could significantly increase the reducing power of WP, and as the DH increases, the reducing power increased slowly. After the *in vitro* simulated gastrointestinal digestion, the reducing power of WPH-GI showed no significant change compared to WPH, indicating that the papain hydrolysate has good digestion stability. Moreover, no significant difference (*p* > 0.05) was observed on the reducing power of the WPH-GI with the DH of 4 and 8% compared with that of WP-GI. The reducing power of WPH-GI produced by flavourzyme (Figure 4E) was limited due to the low enzymatic hydrolysis efficiency. After the simulated digestion *in vitro*, the reducing power of WPH-GI was greatly improved, but it was still lower than that of WP-GI (*p* < 0.05).

In summary, the results of enzymatic hydrolysis of the five different proteases showed that all of the proteases could hydrolyze WP into WPH with DPPH radical-scavenging activity and reducing power, but the enzymatic hydrolysis efficiency of different proteases and the antioxidant activities of WPH at the same DH are different. In addition, WP could be hydrolyzed by pepsin and pancreatin in the simulated digestion system to exert antioxidant activities. Although the process of enzymatic hydrolysis could obtain bioactive peptides with high antioxidant capacity properties, their activities not increase significantly or even decrease after *in vitro* simulated digestion. This is due to the unique cleavage sites of gastrointestinal digestive enzymes. As a kind of endopeptidase, pepsin mainly catalyzes the decomposition of acidic amino acid carboxyl terminal peptide bonds to form peptides, changes the natural conformation of the intact proteins, and facilitates the further digestion of pancreatin (16). Pancreatin is a mixture of multiple enzymes, including trypsin, chymotrypsin, elastase and carboxypeptidase, which function jointly to cleave peptides into oligopeptides and amino acids with certain biological activity (17). Trypsin mainly catalyzes the decomposition of the C-terminal peptide bonds of basic amino acids residues, especially the peptide bonds adjacent to Arg and Lys. Chymotrypsin decomposes peptide bonds adjacent to hydrophobic amino acids and aromatic amino acid residues (14). Elastase catalyzes the decomposition of peptide bonds at the C-terminus of short-chain fatty chains, including Ala, Gly, Ser etc. Additionally, carboxypeptidase is a kind of exopeptidase that systematically removes amino acids from the C-terminus of peptides produced by digestion with other endopeptidases (18), amongst, carboxypeptidase A preferentially releases Val, Leu, Ile, Ala, and carboxypeptidase B releases the basic amino acids Arg and Lys (17). Our previous study has found that WP has a high content of Arg (14.27%), Leu (6.75%), and Vel (4.55%), as well as acidic amino acids (33.66%) and hydrophobic amino acids (27.18%), which results in a good digestibility of WP (10). Antioxidant capacity properties of peptides are partly attributed to the amino acid composition (19). He et al. (20) reported that hydrophobic amino acids (such as Leu, Ile, Pro, Tyr and Met) endow protein hydrolysates with antioxidant capacity due to their abundant electrons can be donated to quench free radicals. Aromatic amino acids (Tyr, Phe) have been considered to enhance the potency of radical scavenging ability by donating protons to stabilize electron-deficient radicals, and maintain their stability through resonance structures (21). Therefore, after the evaluation of antioxidant activity in two-stages of *in vitro* limited enzymolysis and *in vitro* simulated gastrointestinal digestion, it was found that the DH of 0% for limited enzymolysis might be the best choice for exerting its antioxidant activity. That means, WP can exert its potential antioxidant activities after being digested by pepsin and pancreatin in human body without additional enzymatic hydrolysis by commercial protease. To further confirm this result, WP (WPH with the DH of 0%) and its alcalase hydrolysate with DH of 8% was selected for *in vivo* verification experiments.

### Weight Growth Rate and Organ Index of SD Rats During the *in vivo* Antioxidant Test

The weight growth rate and organ index of SD rats reflect the degree of oxidative damage caused by D-galactose to the rat body, and also reflect the protective effect of WP and WPH on oxidative damage. As depicted in Table 2, the weight growth rate of the D-Gal group was significantly (*p* < 0.01) lower than that of the control group, which is consistent with the findings of Li et al. (22). The weight growth rate of rats in the WP-I, WP-II and WPH groups was higher than that of the D-Gal group, but still significantly lower than that of the control group (*p* < 0.05), especially the WP-II group (*p* < 0.01). The WPH group showed the most significant improvement in weight loss caused by oxidative damage. The results of weight growth rate indicated that the injection of D-galactose had a certain inhibitory effect on the weight gain of SD rats, while the intragastric administration of WP and WPH could alleviate the slow weight gain of rats caused by oxidative damage.

**Table 2:** Effects of walnut protein and its hydrolysate on weight gain and organ index of SD rats.

**Group**	**Weight growth rate (%)**	**Organ indexes (%)**				
		**Heart**	**Liver**	**Spleen**	**Kidney**	**Lung**
Control	131.247 ± 12.754	0.314 ± 0.031	2.938 ± 0.217	0.193 ± 0.010	0.662 ± 0.057	0.364 ± 0.062
D-Gal	106.959 ± 7.309^**^	0.282 ± 0.025^*^	2.682 ± 0.236^**^	0.167 ± 0.037^*^	0.636 ± 0.037	0.366 ± 0.041
WP-I	115.611 ± 8.624^*^	0.295 ± 0.023	2.804 ± 0.151	0.175 ± 0.026	0.654 ± 0.053	0.360 ± 0.053
WP-II	113.373 ± 7.028^**^	0.308 ± 0.029	2.856 ± 0.235	0.190 ± 0.044	0.645 ± 0.041	0.369 ± 0.021
WPH	117.425 ± 13.017^*^	0.302 ± 0.028	2.833 ± 0.167	0.196 ± 0.031	0.669 ± 0.070	0.381 ± 0.044

*Control represents the normal control group (saline); D-Gal represents the model control group (D-galactose); WP-I and WP-II represents the low dosage and high dosage of walnut protein group, respectively; WPH represents the walnut protein hydrolysate group*.

* *Significant differences (p < 0.05) and*

** *significant differences (p < 0.01) vs. the control group, respectively*.

The organ index of SD rats is shown in Table 2. It can be seen that the organ index of the liver, heart and spleen tissue of rats in the D-Gal group was significantly lower than that of the control group (*p* < 0.05), especially the liver tissue (*p* < 0.01), whereas no significant change in any organ index was observed in the WP-I, WP-II and WPH groups compared with the control group (*p* > 0.05). This results indicated that the injection of D-galactose had certain damage to the liver, heart and spleen of SD rats, while administration of WP and WPH could effective relieve the oxidative stress and enhance antioxidant defense in the body. Li et al. (22) also found that the injection of D-galactose caused liver and kidney atrophy, and tilapia skin collagen polypeptides treatment could improve the decline of organ function in mice caused by D-galactose.

### Histopathology Analysis of the Liver Tissues

**Figure 5:**
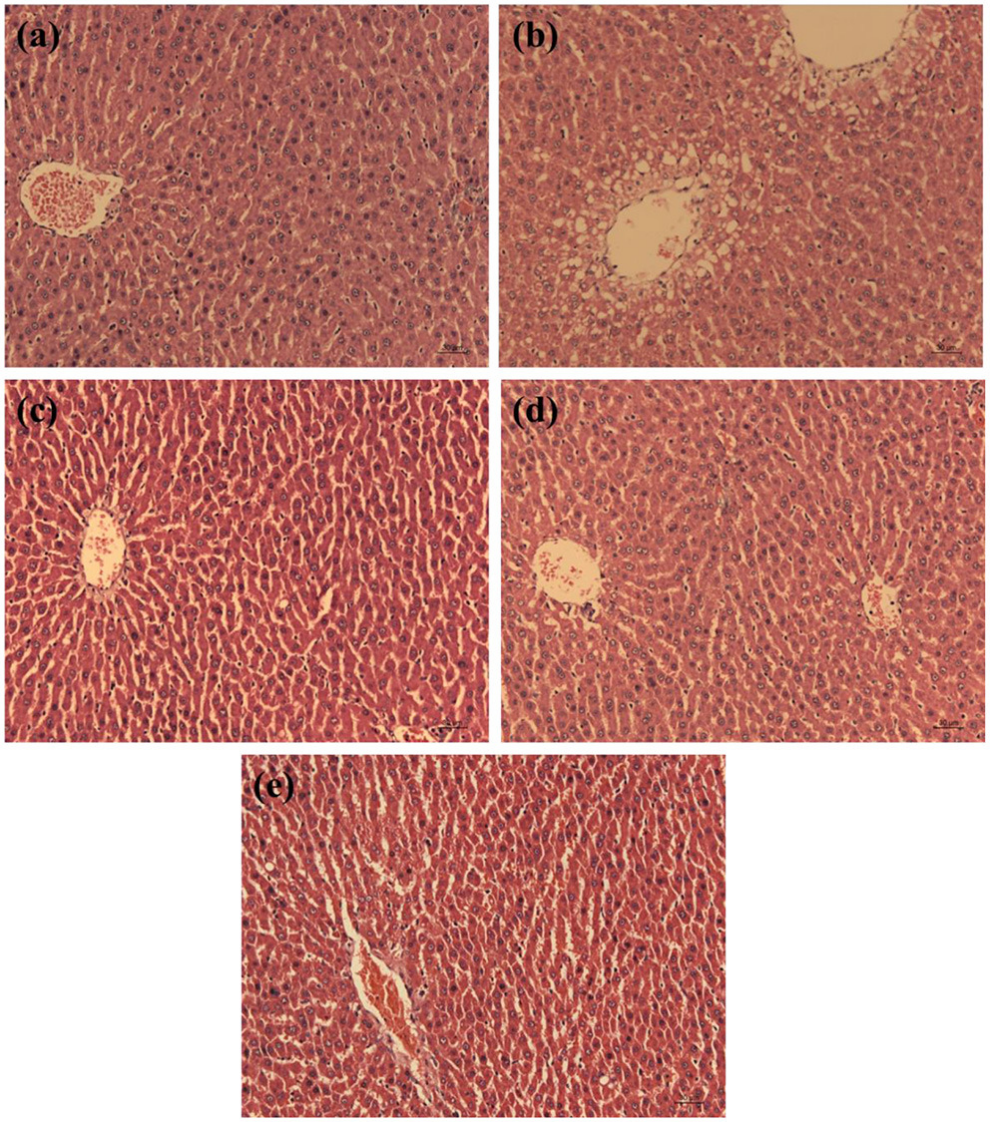
Effect of walnut protein and its hydrolysate on liver histology in D-galactose-treated rats. **(a)** Control group; **(b)** D- galactose group; **(c)** Walnut protein of low dose group; **(d)** Walnut protein of high dose group; **(e)** Walnut protein hydrolysate group.

An oversupply of D-galactose can cause the tissue damage in rats, especially in the liver (22). The histopathology of liver tissue was observed and the results are showed in Figure 5. In the control group, the hepatocytes of rats were in normal shape with regular cell shapes and structurally sound hepatic lobule, radially arranged hepatic cords around a central vein, prominent nuclei and nucleoli, and uniform and dense arrangement. Conversely, compared with the control group, the rats in the D-Gal group had lesions around the central vein area of the liver, the liver cells were enlarged with unclear borders, the cords were arranged disorderly, and the hepatic lobule structure was destroyed. Nevertheless, compared with the D-Gal group, the structure and morphology of hepatocytes in the three treatment groups were ameliorated gradually, the hepatic cords were arranged relatively neatly. Both WP and WPH exhibited a preventive effect against D-galactose-induced pathologic damage to the liver.

### Serum Biochemical Indexes of SD Rats in a Long-Term Experiment

**Figure 6:**
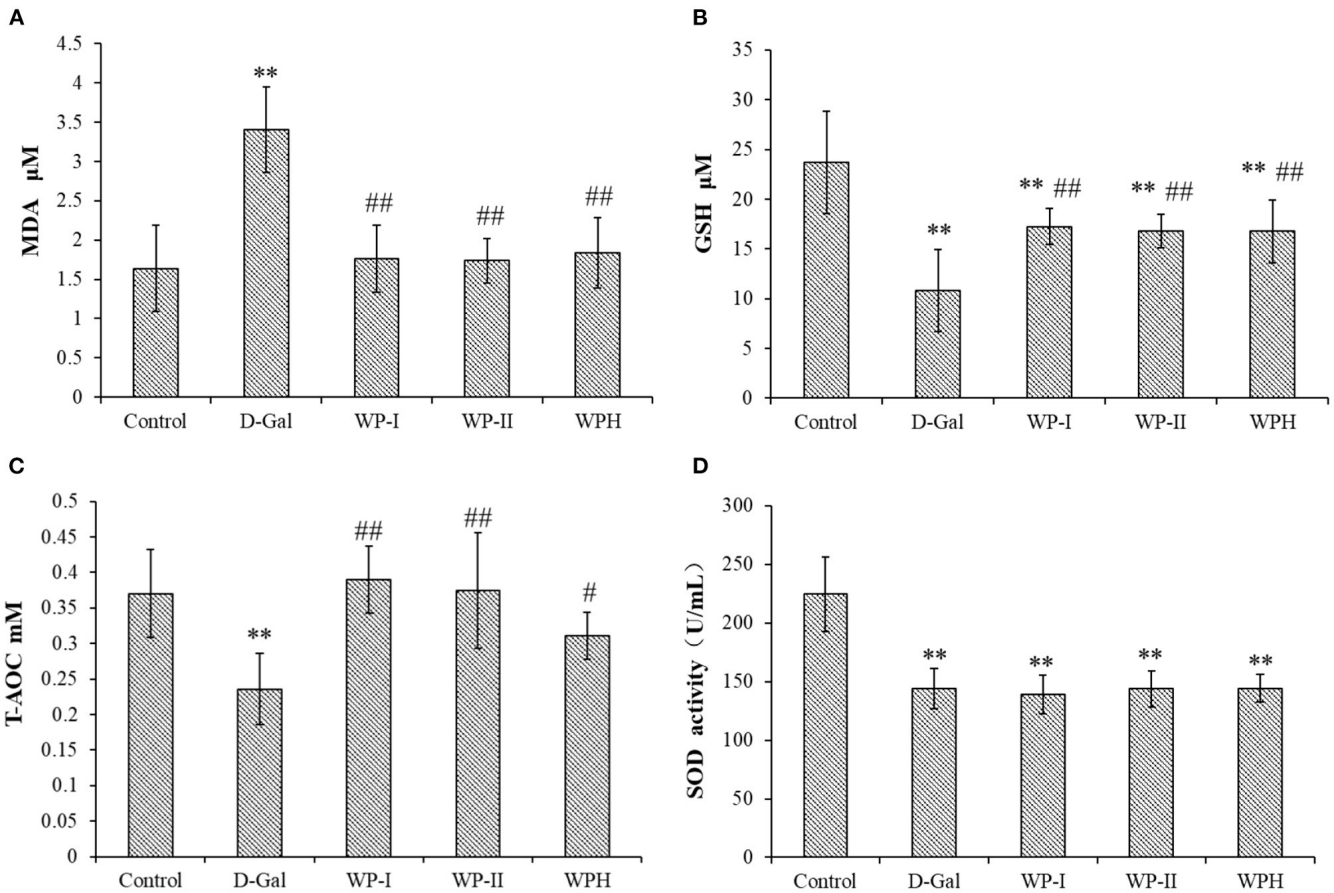
Effect of walnut protein and its hydrolysate on serum biochemical indexes in D-galactose-induced oxidative damage rats. **(A)** MDA; **(B)** GSH; **(C)** T-AOC and **(D)** SOD. **means significantly different (*p* < 0.01) compared to the control group. ^#^significant differences (*p* < 0.05) and ^*##*^significant differences (*p* < 0.01) vs. the D-Gal group, respectively. Control represents the normal control group (saline); D-Gal represents the model control group (D-galactose); WP-I and WP-II represents the low dosage and high dosage of walnut protein group, respectively; WPH represents the walnut protein hydrolysate group.

To further explore the antioxidant effect of WP and WPH in the body, the MDA and GSH contents and activities of antioxidant enzymes SOD and T-AOC in serum of rats were measured, and the results are presented in Figure 6. As a product of polyunsaturated fatty acid peroxidation, MDA is one of an reactive carbonyl compound decomposed by the unstable lipid peroxides (23). MDA content often reflects the lipid peroxidation level *in vivo*, and indirectly reflects the degree of oxidative damage of cells (24). As depicted in Figure 6A, the content of MDA in the serum of rats in the D-Gal group was 2.07 times higher than that of the control group (*p* < 0.01), indicating that the D-galactose-induced oxidative stress model of SD rats was successfully established. The treatment with WP and WPH could significantly reduce the MDA level in serum, and there was no significant difference in MDA level among WP-I, WP-II and WPH groups (*p* > 0.05). These results indicate that administration of WP and WPH can effective reduce lipid peroxidation as seen in lowered MDA content, thereby reducing the oxidative damage caused by D-galactose. In addition, there was no significant difference (*p* > 0.05) in the inhibition of lipid peroxidation between WP and WPH. GSH is an important non-enzymatic antioxidant in the body (25). From Figure 6B, it can be seen that the GSH level was significantly (*p* < 0.01) decreased by D-Gal group (54.53%) compared with the control group, indicating that the oxidative stress model was successfully constructed. In comparison with the control group, the GSH levels of WP-I, WP-II and WPH groups also showed a significant (*p* < 0.01) reduction by 27.39, 29.30, and 29.30%, respectively, while the GSH levels of these three treatment groups were significantly higher (*p* < 0.01) than the D-Gal group. Moreover, no significant difference was observed on the GSH level amongst the WP-I, WP-II and WPH groups (*p* > 0.05). These results suggest that both WP and WPH can effectively alleviate the decrease of GSH level caused by D-galactose-induced oxidative damage, thereby enhancing antioxidant defense, reducing oxidative stress and protect rats from oxidative damages.

Similar to the MDA and GSH level, D-galactose treatment also successfully and significantly reduced (*p* < 0.01) the activities of the innate antioxidant enzymes SOD (Figure 6D) and non-enzymatic antioxidant marker T-AOC (Figure 6C) in the serum of rats in the D-Gal group when compared to the control group. However, the SOD activities of the three treatment groups were not significantly different from that of the D-Gal group (*p* > 0.05), and was significantly lower than that of the control group (*p* < 0.01). T-AOC is an index that comprehensively react the antioxidant enzyme system of the body to resist external oxidative damage and scavenge radicals, reflecting the overall antioxidant capacity (23). As shown in Figure 6C, in comparison with the D-Gal group, the low-dose and high-dose WP treatment showed a significant improvement (*p* < 0.01) on T-AOC level in the serum of rats, as well as the WPH treatment (*p* < 0.05). Taken together, the results of *in vivo* antioxidant tests demonstrated that D-galactose could disrupt the antioxidant defense system of rats, cause oxidative damage and promote lipid peroxidation, while WP and WPH treatment could prevent these damages, and the antioxidant effect of WP was more significant than that of WPH. The protective effect of WP and WPH against oxidative damage might be rationalized due to the impairment of antioxidant defenses systems damage.

## Conclusions

This study established a two-stage enzymatic hydrolysis method based on *in vitro* limited enzymatic hydrolysis and *in vivo* gastrointestinal digestion, and applied it to evaluate the antioxidant activity exerted from WP. Additionally, D-galactose induced oxidative damage model of SD rats was used to verify it. The results of *in vitro* limited enzymatic hydrolysis demonstrated that the optimal DH of *in vitro* limited enzymolysis of WP was 0%, that means WP could be well-digested by pepsin and pancreatin to exert potential antioxidant activities without hydrolysis by commercial proteases. *In vivo* experiment results showed that both WP and WPH could effectively alleviate the oxidative stress and enhance antioxidant defense in the body and exhibited a preventive effect against D-galactose-induced oxidative damage. In short, WP can be a potential food source to exert antioxidant activities directly without *in vitro* enzymatic hydrolysis.

## Data Availability Statement

The original contributions presented in the study are included in the article/supplementary material, further inquiries can be directed to the corresponding author.

## Ethics Statement

The animal study was reviewed and approved by the Institutional Animal Care and Use Committee of Jiangsu University (UJS IACUC).

## Author Contributions

DL: conceptualization, formal analysis, and writing—original draft. MC: methodology and investigation. JZ and WT: investigation and visualization. YG: writing—review and editing. HM: conceptualization, resources, and supervision. All authors contributed to the article and approved the submitted version.

## Funding

The authors gratefully acknowledge the financial support provided by the National Programs for High Technology Research and Development of China grant (grant 2013AA102203).

## Conflict of Interest

The authors declare that the research was conducted in the absence of any commercial or financial relationships that could be construed as a potential conflict of interest.

## Publisher's Note

All claims expressed in this article are solely those of the authors and do not necessarily represent those of their affiliated organizations, or those of the publisher, the editors and the reviewers. Any product that may be evaluated in this article, or claim that may be made by its manufacturer, is not guaranteed or endorsed by the publisher.
